# Impact of Age-Adjusted Charlson Comorbidity on Hospital Survival and Short-Term Outcome of Patients with Extracorporeal Cardiopulmonary Resuscitation

**DOI:** 10.3390/jcm7100313

**Published:** 2018-09-29

**Authors:** Li-Jung Tseng, Hsi-Yu Yu, Chih-Hsien Wang, Nai-Hsin Chi, Shu-Chien Huang, Heng-Wen Chou, Hsin-Chin Shih, Nai-Kuan Chou, Yih-Sharng Chen

**Affiliations:** 1 Institute of Emergency and Critical Care Medicine, National Yang-Ming University, Taipei 11221, Taiwan; ntuhtlj@gmail.com (L.-J.T.); hcshih@vghtpe.gov.tw (H.-C.S.); 2 Department of Surgery, National Taiwan University Hospital, and College of Medicine, National Taiwan University, No.7, Chung-Shan South Road, Taipei 10002, Taiwan; wchemail@gmail.com (C.-H.W.); chinaihsin@gmail.com (N.-H.C.); dtsurg99@yahoo.com.tw (S.-C.H.); ntuhcvs@gmail.com (H.-W.C.); nick.chounaikuan@gmail.com (N.-K.C.); yschen1234@gmail.com (Y.-S.C.)

**Keywords:** extracorporeal membrane oxygenation, cardiopulmonary resuscitation, extracorporeal cardiopulmonary resuscitation, Charlson comorbidity index, age-adjusted Charlson comorbidity index

## Abstract

Extracorporeal cardiopulmonary resuscitation (ECPR) has gradually come to be regarded as an effective therapy, but the hospital mortality rate after ECPR is still high and unpredictable. The present study tested whether age-adjusted Charlson comorbidity index (ACCI) can be used as an objective selection criterion to ensure the most efficient utilization of medical resources. Adult patients (age ≥ 18 years) receiving ECPR at our institution between 2006 and 2015 were included. Data regarding ECPR events and ACCI characteristics were collected immediately after the extracorporeal membrane oxygenation (ECMO) setup. Adverse events during hospitalization were also prospectively collected. The primary endpoint was survival to hospital discharge. The second endpoint was the short-term (2-year) follow-up outcome. A total of 461 patients included in the study were grouped into low ACCI (ACCI 0–3) (240, 52.1%) and high ACCI (ACCI 4–13) (221, 47.9%) groups. The median ACCI was 2 (interquartile range (IQR): 1–3) and 5 (IQR: 4–7) for the low and high ACCI groups, respectively. Cardiopulmonary resuscitation (CPR)-to-ECMO duration was comparable between the groups (42.1 ± 25.6 and 41.3 ± 20.7 min in the low and high ACCI groups, respectively; *p* = 0.754). Regarding the hospital survival rate, 256 patients (55.5%) died on ECMO support. A total of 205 patients (44.5%) were successfully weaned off ECMO, but only 138 patients (29.9%) survived to hospital discharge (32.1% and 27.6% in low and high ACCI group, *p* = 0.291). Multivariate logistic regression analysis revealed CPR duration before ECMO run (CPR-to-ECMO duration) and a CPR cause of septic shock to be significant risk factors for hospital survival after ECPR (*p* = 0.043 and 0.014, respectively), whereas age and ACCI were not (*p* = 0.334 and 0.164, respectively). The 2-year survival rate after hospital discharge for the 138 hospital survivors was 96% and 74% in the low and high ACCI groups, respectively (*p* = 0.002). High ACCI before ECPR does not predict a poor outcome of hospital survival. Therefore, ECPR should not be rejected solely due to high ACCI. However, high ACCI in hospital survivors is associated with a higher 2-year mortality rate than low ACCI, and patients with high ACCI should be closely followed up.

## 1. Background

Extracorporeal membrane oxygenation (ECMO) rescue under cardiopulmonary resuscitation (CPR) can dramatically increase the survival rates of patients who are previously considered to be unsavable. Extracorporeal CPR (ECPR) has been regarded an effective adjuvant therapy [1,2,3]. An increasing number of medical institutes have incorporated this procedure into their CPR protocol [4,5,6,7]. However, the hospital mortality rate among patients who receive ECPR is still high [8], and it is unpredictable because of heterogeneous etiologies, clinical conditions before ECPR, underlying characteristics, and patient selection bias [9]. Objective selection criteria are mandatory to avoid unnecessary use of ECMO, and to focus limited medical resources on the patients with the highest possible chance of survival.

Risk scores have been developed for a variety of acute and chronic disease situations. Among them, the Charlson comorbidity index (CCI) [10] and age-adjusted CCI (ACCI) [11] have been repeatedly validated for estimating the longitudinal prognosis associated with multiple pre-existing comorbidities [12,13]. The ACCI has also been validated [14] in clinical scenarios, such as cancer [15,16], heart failure [17], infectious disease, emergency surgery [18], intensive care units (ICUs), and even the cost-effectiveness analysis of health care systems [13,19]. We considered whether the ACCI could be used as a useful inclusion/exclusion criterion for patients with ECPR.

In addition, little is known regarding the short-term and long-term outcomes and quality of life in patients who have survived after ECPR. To our knowledge, no information regarding the predictive power of the ACCI on follow-up survival for patients who receive ECPR has been published. We conducted the present study to address these issues.

## 2. Methods

**Figure 1:**
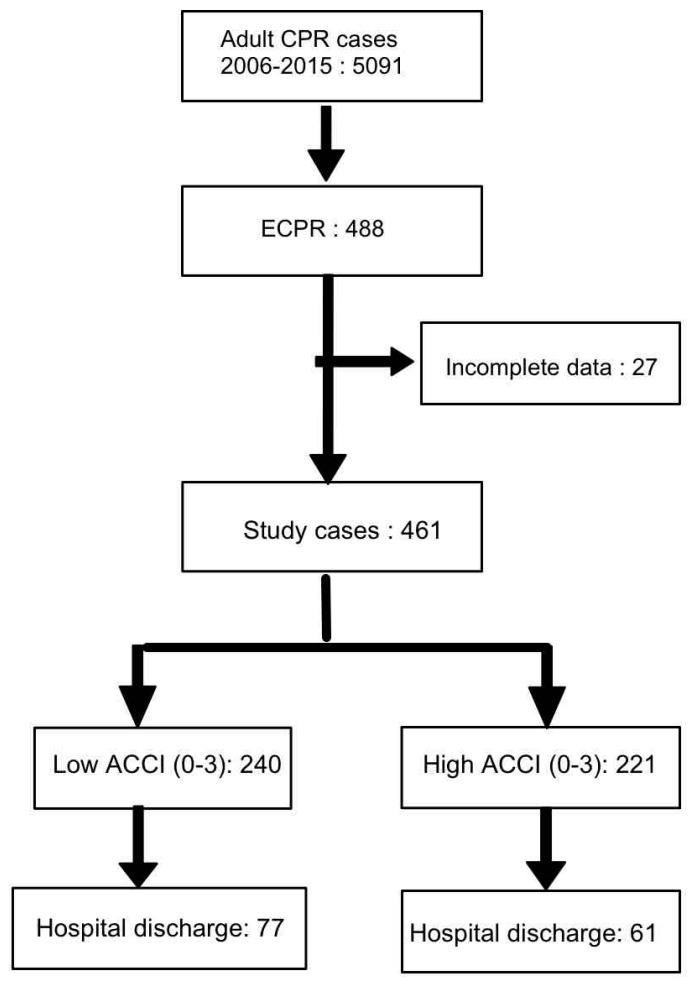
Flowchart of study patients. CPR: cardiopulmonary resuscitation; ECPR: extracorporeal cardiopulmonary resuscitation; ACCI: age-adjusted Charlson comorbidity index.

Our institute started our ECMO program in 1994 and established a task force committee for ECPR in 2003 [1,2]. All data have been prospectively collected and systemically reviewed since 2003. All adult patients (age ≥ 18 years) receiving ECPR at our institution between 2006 and 2015 were included in this study. The institutional review board approved the present study (NTUH 201603049DIPA). Part of the data was reported in previous studies [2,3]. A CPR event was defined as the documented loss of a pulse and respiration with the patient receiving multiple doses of epinephrine injection or undergoing chest compression with or without defibrillation. The CPR duration was defined as the interval between the initiation of CPR and the initiation of ECMO support. Patients experiencing shock necessitating ECMO in an elective condition or an emergency situation without cardiac massage or multiple boluses of epinephrine injection were excluded. Patients with a CPR duration of <10 min were excluded because they did not fit the criterion of “prolonged” CPR. The flow chart diagram is presented in Figure 1.

The equipment and management of ECMO has been described in previous studies [1,2]. The principal components of the ECMO circuit employed with our patients were a centrifugal pump and an oxygenator (Medtronic, Anaheim, CA, USA; Medos, Stolberg, Germany; Maquet, Rastatt, Germany). The circuit was preorganized without priming, and it was primed with saline containing 2 U/mL heparin when the ECPR call was initiated. The equipment and instruments required for cannulation were preconfigured on a cart for rapid response in an ECPR situation [3].

Out-of-hospital cardiac arrest (OHCA) was defined as any cardiac arrest outside the hospital. For OHCA patients in cardiac arrest, a rapid primary survey, followed by CPR and attempted defibrillation was initiated by well-trained emergency medical technicians complying with the guidelines of the American Heart Association [20]. Patients were transported to our institution with CPR being performed continually during transportation. Upon arrival at the emergency department, if the patient failed to achieve return of spontaneous circulation after administration of two doses of epinephrine and advanced cardiovascular life support (ACLS) for 10 min, the physicians consulted the ECMO team to decide whether the patient was eligible for ECPR.

The relative and absolute contraindications against ECMO use during CPR emergency consultation were reported in a previous study [3] and are summarized in Table A1. The unfavorable conditions for ECMO support during CPR included age over 75 years (relative), knowledge of severe irreversible brain damage before CPR, terminal malignancy without reversible iatrogenic conditions, a traumatic origin with uncontrolled bleeding, and those who had signed “do not attempt resuscitation” orders.

The patients’ demographic and health data before hospitalization were collected after ECMO setup. Adverse events during hospitalization were also collected prospectively during hospitalization. The CCI before CPR was calculated according to a reported formula containing 17 variables [14,21]. The ACCI equaled the CCI plus 1 point for each decade over age 40, up to 5 points for patients >80 years [14,15]. Medical events before ECPR (e.g., acute myocardial infarct, congestive heart failure, and respiratory failure) and after ECPR (e.g., new-onset renal failure requiring long-term dialysis, stroke, and hemiplegia) were retrieved from our ECMO database. The ACCI was recalculated at hospital discharge for post-ECPR survivors.

For patients who survived after ECPR, the follow-up period was a minimum of two years (until 31 December 2017). Follow-up status and events were collected using a chart review and a telephone interview. The primary endpoint of the present study was survival to hospital discharge. The second endpoint was a short-term (2-year) follow-up outcome.

Statistics: Continuous variables with a normal distribution are presented as the mean ± standard deviation (SD), or with 95% confidence intervals (CIs), and were examined using Student’s t-tests. Continuous variables with a skewed distribution were presented as the median and 25–75% interquartile range, or with 95% CIs, and they were examined using the Mann–Whitney *U* test (unpaired comparison) or the Wilcoxon test (paired comparison). Categorical variables were analyzed using chi-squared (*χ^2^*) tests. For significant variables of adverse events, variables with *p* < 0.15 through univariate analysis underwent multivariate logistic regression with backward stepwise analysis. Hazard ratios (HRs) of each significant variable were calculated by either univariate or multivariate logistic regression. Survival was modeled using the Kaplan–Meier method, and the log-rank test was applied for statistical significance. Statistical significance was set at *p* < 0.05. All statistical analyses were performed using MedCalc Statistical Software version 17.8.6 (MedCalc Software, Ostend, Belgium).

## 3. Results

**Figure 2:**
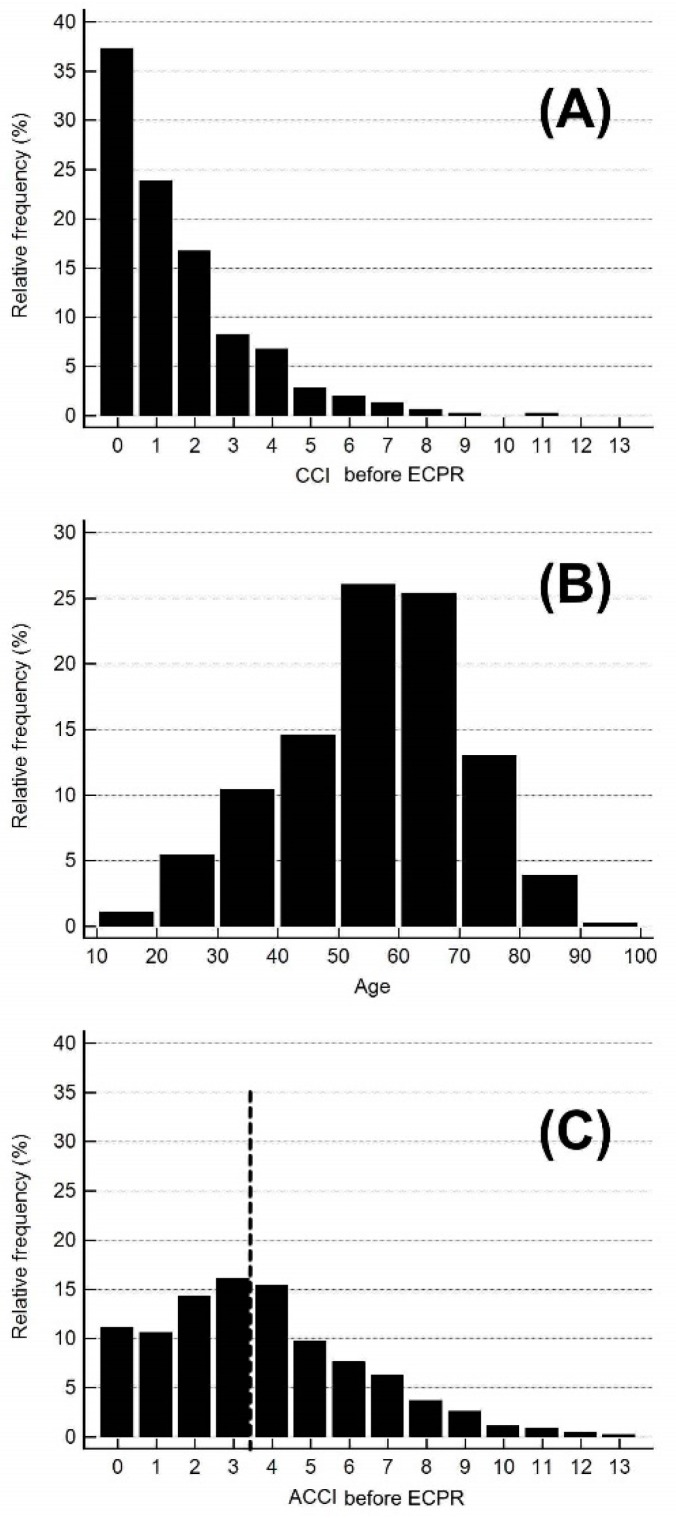
Histogram of the (**A**) Charlson comorbidity index (CCI); (**B**) age; and (**C**) age-adjusted Charlson comorbidity index (ACCI) for the study patients. ECPR: extracorporeal cardiopulmonary resuscitation.

A total of 461 adult patients receiving ECPR in our hospital during 2006 to 2015 were included in the study. Figure 2 demonstrates the histogram of the study patients. In brief, the CCI before ECPR ranged from 0 to 11 with a mean of 1.53 ± 1.81. The age ranged from 19 to 95 years, with a mean of 55.5 ± 15.3 years. The ACCI ranged from 0 to 13, with a mean of 3.69 ± 2.62. According to the histogram of ACCI, the study patients were grouped into low ACCI (ACCI 0–3) and high ACCI (4–13) groups (accounting for 52.1% and 47.9%, respectively). Their demographic data are presented in Table 1. Regarding the individual variables in the CCI, all variables except two (autoimmune disease and acquired immune deficiency syndrome (AIDS)) were more frequent in the high ACCI group than in the low ACCI group (*p* < 0.05 for all variables except autoimmune disease and AIDS). 

Regarding the main causes of CPR, acute coronary syndrome (ACS) was the leading cause, with 41.9% of patients, followed by chronic heart failure (13.9%), septic shock (7.2%), and postcardiotomy shock (6.7%) (Table 1). The CPR duration before ECMO support (CPR-to-ECMO duration) was comparable between the groups (42.1 ± 25.6 min in the low ACCI group and 41.3 ± 20.7 min in the high ACCI group, *p* = 0.754). OHCAs were more common in the low ACCI group (24.6%) than in the high ACCI group (13.6%) (*p* = 0.003), possibly reflecting a clinical judgment bias in OHCA against relatively old and frail patients.

Table 2 lists the major mortality and morbidity events after ECPR and ECMO. The numbers of ECMO support, ventilator support, ICU, and hospitalization days were 4.79 ± 17.53, 11.1 ± 19.30, 12.7 ± 18.57, and 22.0 ± 34.4 days, respectively. These medical utilization days did not differ between the low and high ACCI groups (Table 2).

Regarding the hospital mortality rate after ECPR and ECMO support, 256 patients (55.5%) died on ECMO support. A total of 205 patients (44.5%) were successfully weaned off ECMO, but 67 of them (14.6%) died during hospitalization, including heart failure (23), sepsis (19), unrecovered consciousness (11), cerebral vascular attack (CVA) (4), respiratory failure (3), malignancy terminal stage (3), miscellaneous (4). The remaining 138 patients (29.9%) survived to hospital discharge. The hospital discharge rate was not different between the low and high ACCI groups (32.1% and 27.6%, respectively; *p* = 0.294).

As shown in Table 2, the percentage of post-ECPR morbidity was high, with vascular, neurologic, and renal complications in 6.7%, 41.9%, and 44.3% of patients, respectively. These post-ECPR complications were not significantly different between the low and high ACCI groups, except for post-ECPR renal failure (38.7% vs. 50.2% for the low and high ACCI groups, respectively; *p* = 0.013).

**Figure A1:**
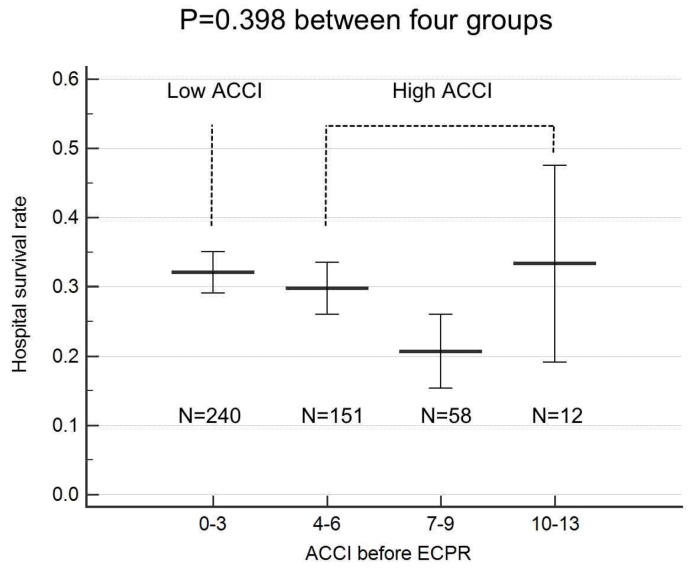
Hospital survival rate grouped by age-adjusted Charlson comorbidity index (ACCI) before extracorporeal cardiopulmonary resuscitation (ECPR).

Univariate and multivariate logistic regression analyses for risk factors in hospital survival after ECPR are listed in Table 3. Only CPR-to-ECMO duration, CPR cause of ACS, and CPR cause of septic shock were significant risk factors in the hospital survival rate. In contrast, CCI, age, and ACCI were not risk factors for hospital survival after ECPR (*p* = 0.205, 0.334, and 0.164, respectively). The hospital survival rate was comparable for the ACCI 0–3, 4–6, 7–9, and 10–13 groups, respectively (*p* = 0.398; Figure A1). In multivariate logistic regression analysis, only CPR duration before ECMO run and CPR cause of septic shock were significant risk factors (*p* = 0.043 and 0.014, respectively).

**Figure 3:**
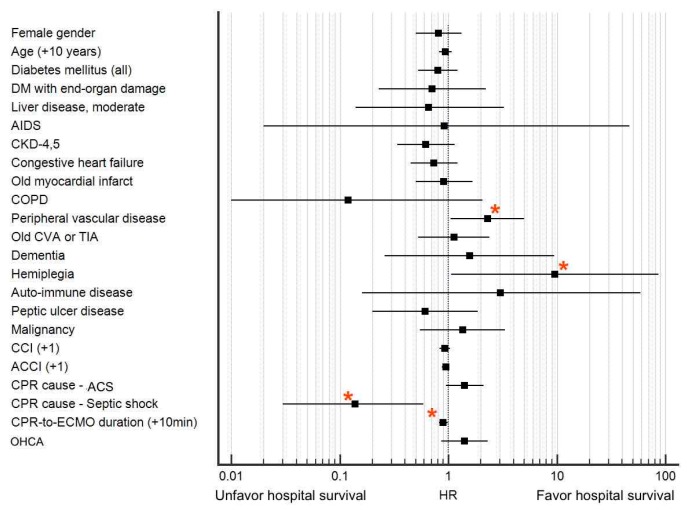
Hazard ratio (HR) of individual factors for hospital survival. Four significant risk factors are marked with red asterisks, namely peripheral vascular disease (HR = 2.3, 95% confidence interval (CI) = 1.05–5.02, *p* = 0.037), hemiplegia (HR = 9.6, 95% CI = 1.06–86.8, *p* = 0.04), cardiopulmonary resuscitation (CPR) due to septic shock (HR = 0.14, 95% CI = 0.03–0.59, *p* = 0.007), and CPR duration before extracorporeal membrane oxygenator (ECMO) run (CPR-to-ECMO duration) (HR = 0.9 for +10 min, 95% CI = 0.82–0.99, *p* = 0.04). Notably, age, Charlson comorbidity index (CCI), and age-adjusted Charlson comorbidity index (ACCI) were not significant risk factors (age: HR = 0.94 for +10 years, 95% CI = 0.82–1.07, *p* = 0.333; CCI: HR = 0.93, 95% CI = 0.83–1.04, *p* = 0.205; and ACCI: HR = 0.95, 95% CI = 0.87–1.02, *p* = 0.164). AIDS: acquired immune deficiency syndrome, CKD: chronic kidney disease, COPD: chronic obstructive pulmonary disease, CVA: cerebral vascular attack, TIA: transient ischemic attack, ACS: acute coronary syndrome, CPR-to-ECMO duration: CPR duration before ECMO run, OHCA: Out-of-hospital cardiac arrest, DM: diabetes mellitus. * Denotes variables with *p* < 0.05 by univariate analysis.

The HR of all variables in the ACCI for the hospital survival rate is plotted in Figure 3. Four variables were significant for hospital survival; two related to CPR conditions (CPR due to septic shock: HR = 0.14, 95% CI = 0.03–0.59, *p* = 0.007 and CPR-to-ECMO duration: HR = 0.9 for +10 min, 95% CI = 0.82–0.99, *p* = 0.04) and two related to underlying conditions (peripheral vascular disease: HR = 2.3, 95% CI = 1.05–5.02, *p* = 0.037 and hemiplegia: HR = 9.6, 95% CI = 1.06–86.8, *p* = 0.04). Notably, age, CCI, and ACCI were not significant risk factors (age: HR = 0.94 for +10 years, 95% CI = 0.82–1.07, *p* = 0.333; CCI: HR = 0.93, 95% CI = 0.83–1.04, *p* = 0.205; and ACCI: HR = 0.95, 95% CI = 0.87–1.02, *p* = 0.164).

**Figure 4:**
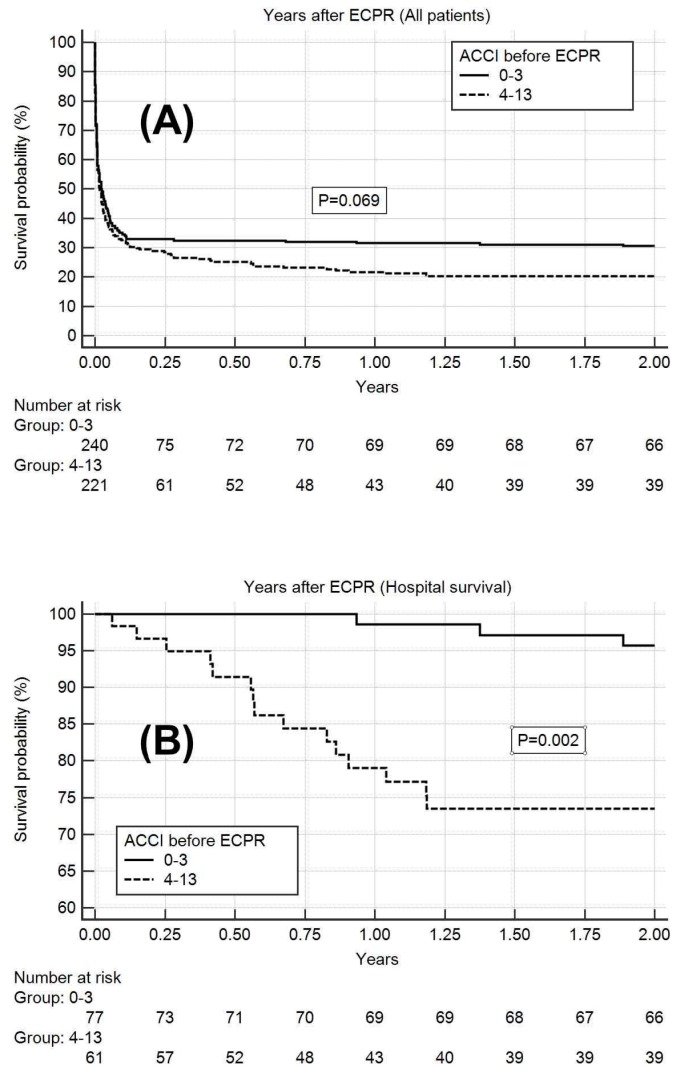
Kaplan–Meier analysis for a 2-year survival rate, with patients grouped by age-adjusted Charlson comorbidity index (ACCI) before extracorporeal cardiopulmonary resuscitation (ECPR). (**A**) Kaplan–Meier survival analysis for all patients, which shows the marginal 2-year survival advantage for ACCI 0–3 patients (0.31, standard error (S.E) of 0.03), compared with that for ACCI 4–13 patients (0.20, S.E of 0.03) (*p* = 0.069); (**B**) Kaplan–Meier survival analysis for hospital survivors, which shows the higher 2-year survival rate for ACCI 0–3 patients (0.96, S.E of 0.02), compared with that for ACCI 4–13 patients (0.74, S.E of 0.06) (*p* = 0.002).

For the 138 hospital survivors, a 2-year follow-up after hospital discharge was completed. A total of 18 patients died within two years, three from the low ACCI group and 15 from the high ACCI group. The causes of death were congestive heart failure (8), respiratory failure (3), trauma (2), infection (2), peritonitis (1), renal failure (1), and malignancy (1). A Kaplan–Meier survival analysis for all the patients with ECPR indicated a marginal 2-year survival advantage for the patients with low ACCI (0.31, standard error (S.E) of 0.03) compared with that for the patients with high ACCI (0.20, S.E of 0.03; *p* = 0.069). For the patients who survived (*n* = 138), the Kaplan–Meier survival analysis indicated a higher 2-year survival rate for the patients with low ACCI (0.96, S.E 0.02) than for the patients with high ACCI (0.74, S.E of 0.06; *p* = 0.002; Figure 4).

## 4. Discussion

To our knowledge, the present study is the first to investigate the ACCI regarding the outcome of ECPR. The study result showed that the ECPR survival rate was similar between patients with high and low ACCI, with the low ACCI group not exhibiting a guaranteed survival-to-discharge advantage over the high ACCI group. Therefore, high ACCI should not be regarded as a contraindication for ECPR candidates. However, among the post-ECPR survivors, high ACCI was associated with a high follow-up mortality rate, indicating that continued medical care is mandatory for this group of patients after hospital discharge.

The CCI was developed in 1987 to measure the burden of comorbid disease, and can efficiently predict long-term mortality [10]. It was modified in 1994 by incorporating age to yield the ACCI, which further improved the predictive power of the index [11]. The CCI and ACCI became increasingly popular in medical research studies between 2005 and 2010, because they can be calculated using health claims data for clinical, epidemiological, or cost-utility studies [12,13,19,22]. The CCI was originally designed to predict mortality or the costs associated with chronic diseases in patients and has been extended to evaluate outcome after heart failure treatment [17] or ICU treatment [23]. However, whether it can be used as a prognostic index for acute care after ECPR has not been previously investigated. The data from the present study indicate that the ACCI may be effective as a predictive model, but only in those surviving after the index ECPR; thus, it is not a satisfactory tool for predicting survival rate in the ECPR. A high ACCI unnecessarily predicts poor outcome after ECPR; therefore, old age alone or high ACCI alone should not be considered an absolute contraindication for ECPR in the scene of CPR.

The present study demonstrated that age is not a risk factor in ECPR outcome. This is in accordance with several previous study results [9,24,25]. Due to limited economic and medical facilities for ECMO support, providing ECMO to every patient in emergent CPR situations is impossible. The traditional ECPR selection criterion is typically aged <65–75 years [3,26,27]. However, according to numerous studies [9,24,25] and the present study, old age should not be regarded as an absolute exclusion criterion for ECPR. Instead, a criterion that excludes the least appropriate candidates for ECMO support during a CPR scenario must be employed. CPR-to-ECMO duration and CPR due to sepsis are two crucial risk factors for poor hospital survival, as demonstrated by the present study and previous research [2,3,6,9].

Table 1 illustrates that some differences in parameters existed between the low and high ACCI groups. ECPR for OHCA was less common in the high ACCI group (13.6%) compared with the low ACCI group (24.6%), implying that selection bias might exist in the referral of OHCA patients as potential candidates for ECMO support. In addition, CPR due to ACS or postcardiotomy was more common in patients with a high ACCI (57.9%), compared with the low ACCI group (40.0%), implying that selection bias for relatively acute conditions also existed when referring patients with OHCA for ECMO support.

As illustrated in Figure 2, only peripheral arterial occlusive disease (PAOD) and hemiplegia were associated with a higher hospital survival rate. Regarding PAOD, a detailed analysis revealed that 14 of 27 (51.9%) of patients with PAOD received ECPR due to ACS, which was higher than the 179 of 434 (41.2%) of patients without PAOD (*p* = 0.05). In addition, ACS was associated with a marginally higher chance of hospital survival in the present study (HR = 1.41, 95% CI = 0.94–2.11, *p* = 0.091; Table 3). Several studies [26,28,29] have demonstrated that ECPR, due to ACS, can achieve an improved outcome if prompt percutaneous coronary intervention can be performed soon after effective ECPR.

Regarding hemiplegia as a significant risk factor, owing to the limited case number (*n* = 5), the significance and cause of this factor in hospital survival requires further evaluation. Considering the excessively high mortality rate after hospital discharge for patients with a high ACCI after ECPR (Figure 3), we have highlighted another challenge that suggests continued attention after ECPR hospital discharge should be mandatory for patients with a high ACCI. Because nearly half of the patients died of cardiac-origin causes, we propose that a new organization or taskforce focusing on multimodality cardiac care may be required for early detection of possible ongoing complications after ECPR. 

## 5. Limitations

First, the study period was nine years, during which several modifications were made in ECPR indication and contraindication, as well as decision making and patient care. Secondly, the number of study patients was low, which may have affected the data analysis. Third, the present study was not a prospective randomized study. Therefore, selection bias based on multiple considerations may have existed [6]. Emergency ECMO consultation for older patients with a high CCI or ACCI and with long resuscitation times or suboptimal CPR quality might have been rejected. Fourth, only 9.6% of CPR patients received ECMO support during the study period, this potential selection bias should be taken into consideration when reading this paper.

## 6. Conclusions

The data of the present study indicate that a high ACCI before ECPR does not predict a low likelihood of hospital survival. Therefore, ECPR should not be rejected solely on the basis of a high ACCI. However, a high ACCI was associated with higher 2-year mortality rate among the hospital survivors than a low ACCI, and patients with a high ACCI should be closely followed up.

## Figures and Tables

**Table 1:** Demographic and clinical data of the study patients grouped by ACCI before the ECPR index.

Variables	All (461)	ACCI 0–3 (240)	ACCI 4–13 (221)	*p* (ACCI 0–3 vs. ACCI 4–13)
Female gender	105 (22.8%)	55 (22.9%)	50 (22.6%)	0.941
Age (years)	55.5 ± 15.3	45.8 ± 12.8	66.1 ± 9.96	<0.001
<40	78 (16.9%)	78 (32.5%)	0 (0.0%)	<0.001
40–49	67 (14.5%)	58 (24.2%)	9 (4.1%)	
50–59	120 (26.0%)	77 (32.1%)	43 (19.5%)	
60–69	117 (25.4%)	27 (11.3%)	90 (40.7%)	
70–79	60 (13.0%)	0 (0.0%)	60 (27.1%)	
>80	19 (4.1%)	0 (0.0%)	19 (8.6%)	
Diabetes mellitus	177 (38.4%)	44 (18.3%)	133 (60.2%)	<0.001
Uncomplicated	160 (34.7%)	44 (18.3%)	116 (52.5%)	<0.001
End-organ damage	17 (3.7%)	0 (0.0%)	17 (7.7%)	<0.001
Liver disease, moderate	9 (2.0%)	0 (0.0%)	9 (4.1%)	0.002
AIDS	0 (0.0%)	0 (0.0%)	0 (0.0%)	0.376
CKD-4,5	68 (14.8%)	6 (2.5%)	62 (28.1%)	<0.001
Congestive heart failure	103 (22.3%)	29 (12.1%)	74 (33.5%)	<0.001
Old myocardial infarct	60 (13.0%)	11 (4.6%)	49 (22.1%)	<0.001
COPD	9 (2.0%)	0 (0.0%)	9 (4.1%)	0.002
Peripheral vascular disease	27 (5.9%)	0 (0.0%)	27 (12.2%)	<0.001
Old CVA or TIA	34 (7.4%)	4 (1.7%)	30 (13.6%)	<0.001
Dementia	5 (1.1%)	0 (0.0%)	5 (2.3%)	0.019
Hemiplegia	5 (1.1%)	0 (0.0%)	5 (2.3%)	0.019
Auto-immune connective disease	3 (0.7%)	3 (1.3%)	0 (0.0%)	0.090
Peptic ulcer disease	19 (4.1%)	3 (1.3%)	16 (7.2%)	0.001
Malignancy	22 (4.8%)	1 (0.4%)	21 (9.5%)	<0.001
CCI	1 (IQR: 0–2)	0 (IQR: 0–1)	2 (IQR: 1–4)	<0.001
ACCI	3 (2–5)	2 (IQR: 1–3)	5 (IQR: 4–7)	<0.001
Main CPR causes				<0.001
ACS	193 (41.9%)	83 (34.6%)	110 (49.8%)	0.001
Chronic heart failure	64 (13.9%)	33 (13.8)	31 (14.0%)	
Septic shock	33 (7.2%)	18 (7.5%)	15 (6.8%)	
Post-cardiotomy	31 (6.7%)	13 (5.4%)	18 (8.1%)	
Pulmonary embolism	25 (5.4%)	12 (5.0%)	13 (5.9%)	
Acute myocarditis	19 (4.1%)	18 (7.5%)	1 (0.5%)	
Arrhythmia	18 (3.9%)	11 (4.6%)	7 (3.2%)	
Cardiac tamponade	8 (1.7%)	2 (0.8%)	6 (2.7%)	
Respiratory failure	16 (3.5%)	13 (5.4%)	3 (1.4%)	
Acute aortic dissection	7 (1.5%)	5 (2.1%)	2(0.9%)	
Hypovolemia	12 (2.6%)	9 (3.7%)	3 (1.4%)	
Acute rejection	9 (2.0%)	5 (2.1%)	4 (1.8%)	
Others	26 (5.6%)	18 (7.5%)	8 (3.6%)	
CPR duration before ECMO run (min)	41.7 ± 23.2	42.1 ± 25.6	41.3 ± 20.7	0.754
OHCA	89 (19.3%)	59 (24.6%)	30 (13.6%)	0.003

CCI: Charlson comorbidity index, ACCI: age-adjusted Charlson comorbidity index, AIDS: acquired immune deficiency syndrome, CKD: chronic kidney disease, COPD: chronic obstructive pulmonary disease, CVA: cerebral vascular attack, TIA: transient ischemic attack, ACS: acute coronary syndrome, CPR: cardiopulmonary resuscitation, ECMO: extracorporeal membrane oxygenator, OHCA: Out-of-hospital cardiac arrest, CPR-to-ECMO duration: CPR duration before ECMO run, ECPR: extracorporeal cardiopulmonary resuscitation, IQR: interquartile range.

**Table 2:** Outcome analysis of ECPR. Data are presented for all patients and grouped by ACCI before ECPR.

Variables	All (461)	ACCI 0–3 (240)	ACCI 4–13 (221)	*p*
ECMO days	4.79 ± 17.53	4.48 ± 6.10	5.13 ± 24.5	0.694
Ventilator days	11.1 ± 19.30	11.6 ± 21.2	10.6 ± 17.1	0.547
ICU days	12.7 ± 18.57	12.5 ± 18.5	13.0 ± 18.7	0.780
Hospitalization days	22.0 ± 34.4	21.7 ± 36.1	22.2 ± 32.5	0.899
Mortality				0.069
Die on ECMO	256 (55.5%)	136 (56.7%)	120 (54.3%)	
Weaned off ECMO and die	67 (14.6%)	27 (11.3%)	40 (18.1%)	
Hospital discharge	138 (29.9%)	77 (32.1%)	61 (27.6%)	0.294
Morbidity				
Vascular complications	31 (6.7%)	16 (6.7%)	15 (6.8%)	0.959
Neurological complications	193 (41.9%)	101 (42.1%)	92 (41.6%)	0.921
New renal failure	204 (44.3%)	93 (38.7%)	111 (50.2%)	0.013

ACCI: age-adjusted Charlson comorbidity index, ECMO: extracorporeal membrane oxygenator, ICU: intensive care unit, ECPR: extracorporeal cardiopulmonary resuscitation.

**Table 3:** Logistic regression analysis for hospital survival.

Variables	Univariate Analysis	*p*	Multivariate Analysis	*p*
Female gender	0.81 (0.50–1.32)	0.406	-	-
CCI	0.93 (0.83–1.04)	0.205	-	-
Age (+10 years)	0.94 (0.82–1.07)	0.353	-	-
ACCI	0.95 (0.87–1.02)	0.164	-	-
CPR causes				
ACS	1.41 (0.94–2.11)	0.091 *	1.31 (0.87–1.02)	0.215
Chronic heart failure	0.83 (0.46–1.50)	0.526	-	-
Septic shock	0.14 (0.03–0.59)	0.007 *	0.16 (0.04–0.70)	0.014
Post-cardiotomy	1.31 (0.61–2.82)	0.486	-	-
Pulmonary embolism	1.91 (0.85–4.32)	0.120	-	-
Acute myocarditis	1.76 (0.69–4.44)	0.242	-	-
CPR-to-ECMO duration (+10 min)	0.90 (0.82–1.00)	0.044 *	0.90 (0.81–1.00)	0.043
OHCA	1.41 (0.86–2.30)	0.169	-	-

CCI: Charlson comorbidity index, ACCI: age-adjusted Charlson comorbidity index, ACS: acute coronary syndrome, CPR: cardiopulmonary resuscitation, ECMO: extracorporeal membrane oxygenator, CPR-to-ECMO duration: CPR duration before ECMO run, OHCA: Out-of-hospital cardiac arrest, *: *p* < 0.10.

## Appendix A

**Table A1:** Contraindications of extracorporeal cardiopulmonary resuscitation (ECPR).

Absolute contraindication
Age >90
End-stage malignancy (except reversible adverse reaction due to immuno- or chemotherapy treatment.)
Long-term poor morbidity
Acute intracranial hemorrhage
Uncontrolled bleeding
Prolonged CPR >90 min
Do-not-resuscitation (DNR) prescription
Relative contraindication
Age 75–90
Sepsis
Pre-existing multiple organ failure
Prolonged CPR >60 min
Unwitnessed arrest

CPR: cardiopulmonary resuscitation.
